# Commentary: The impact of dietary inflammation index on benign prostatic hyperplasia: insights from patient data and animal models

**DOI:** 10.3389/fnut.2026.1837515

**Published:** 2026-05-14

**Authors:** Zhihui Li, Lei Wang, Liang Zhong, Dongxiang Zheng

**Affiliations:** 1 The Tenth Clinical Medical College of Guangzhou University of Chinese Medicine, Zhongshan, Guangdong, China; 2 Zhongshan Hospital of Traditional Chinese Medicine, Zhongshan, China

**Keywords:** BPH, dietary inflammatory, letter, Mendelian randomization, NHANES

We read with great interest the recent article by Ke et al. on the association between dietary inflammatory load and benign prostatic hyperplasia (BPH) (1). The topic is clinically relevant, and the integration of NHANES analyses, two-sample Mendelian randomization (MR), and animal experiments is commendable. Such a multi-level design represents a strength of the original study and provides useful hypothesis-generating evidence for the potential relationship between diet-related factors and BPH. Nevertheless, we believe that the causal wording used throughout the manuscript may be stronger than the presented evidence can support.

First, the MR analysis does not appear to instrument the dietary inflammation index (DII) itself. Rather, the authors selected nine dietary preference traits and interpreted the resulting associations as genetic evidence for a causal role for dietary inflammatory load in BPH. However, the reported significant signals included an overall healthy diet, beef consumption, and beer or cider consumption, all of which showed inverse associations with BPH risk. This pattern is not straightforwardly aligned with the broader conclusion that pro-inflammatory dietary patterns causally increase BPH risk. Moreover, dietary preference traits may have limited biological specificity and may reflect complex behavioral, environmental, socioeconomic, and cultural determinants rather than the inflammatory potential of diet alone. Potential horizontal pleiotropy and counterintuitive associations further limit causal interpretation. Thus, the MR findings may be more appropriately interpreted as trait-specific dietary signals rather than direct evidence that DII *per se* causes BPH (2).

Second, the animal experiment does not fully disentangle inflammatory dietary load from caloric density, macronutrient composition, and micronutrient composition. The pro-inflammatory diet provided 4.87 kcal/g, compared with 3.02 kcal/g in the control diet and 3.22 kcal/g in the anti-inflammatory diet, and the same group also had the highest body weight and prostate index (1). Under such conditions, the observed prostatic changes may reflect the combined effects of excess energy intake, adiposity-related metabolic alterations, and inflammation, rather than inflammatory load alone (3, 4). In addition, this model may be more accurately described as diet-associated prostatic enlargement rather than a strict model of age-related or clinically defined BPH. The inflammatory readouts are biologically suggestive, but they remain largely descriptive and do not fully establish that inflammation is the independent causal driver of the observed prostatic changes. In addition, the Methods state that data were expressed as mean ± SD, whereas Figure 4 reports mean ± SEMs, which would benefit from clarification (1).

Finally, the NHANES component remains cross-sectional and relies on questionnaire-based ascertainment of BPH. Because DII and BPH status were assessed simultaneously, the temporal direction of the association cannot be determined. Questionnaire-based BPH ascertainment may also introduce misclassification, particularly in the absence of objective clinical indicators such as prostate volume, International Prostate Symptom Score (IPSS), or histological confirmation. In addition, residual confounding may remain, including hormonal status, medication or treatment history, and physical activity. We therefore respectfully suggest that the conclusions may be better framed as supporting an association with biological plausibility, rather than a definitive causal role.

In summary, this study provides interesting, hypothesis-generating evidence linking diet-related factors to BPH. However, moderating the causal interpretation would improve the precision and balance of the manuscript. The main strength of our Commentary is to highlight the need for cautious interpretation of observational, genetic, and experimental evidence. However, our discussion is limited by our reliance on the published data and reported methods of the original study, without access to individual-level NHANES data, GWAS summary details beyond those reported, or raw animal experimental data. Future studies using direct genetic instruments for DII and isocaloric experimental designs may be better positioned to determine whether dietary inflammatory load independently contributes to BPH pathogenesis.
